# Design and Verification of a Novel Perfusion Bioreactor to Evaluate the Performance of a Self-Expanding Stent for Peripheral Artery Applications

**DOI:** 10.3389/fmedt.2022.886458

**Published:** 2022-06-21

**Authors:** Swati Nandan, Jessica Schiavi-Tritz, Rudolf Hellmuth, Craig Dunlop, Ted J. Vaughan, Eimear B. Dolan

**Affiliations:** ^1^Biomedical Engineering and Biomechanics Research Centre (BioMEC), School of Engineering, College of Science and Engineering, National University of Ireland Galway, Galway, Ireland; ^2^Vascular Flow Technology, Dundee, United Kingdom

**Keywords:** peripheral artery disease (PAD), bioreactor design, *in vitro* testbeds, computational fluid dynamics (CFD), self-expanding nitinol stent, hemodynamic forces, neointimal growth

## Abstract

Endovascular stenting presents a promising approach to treat peripheral artery stenosis. However, a significant proportion of patients require secondary interventions due to complications such as in-stent restenosis and late stent thrombosis. Clinical failure of stents is not only attributed to patient factors but also on endothelial cell (EC) injury response, stent deployment techniques, and stent design. Three-dimensional *in vitro* bioreactor systems provide a valuable testbed for endovascular device assessment in a controlled environment replicating hemodynamic flow conditions found *in vivo*. To date, very few studies have verified the design of bioreactors based on applied flow conditions and their impact on wall shear stress, which plays a key role in the development of vascular pathologies. In this study, we develop a computationally informed bioreactor capable of capturing responses of human umbilical vein endothelial cells seeded on silicone tubes subjected to hemodynamic flow conditions and deployment of a self-expanding nitinol stents. Verification of bioreactor design through computational fluid dynamics analysis confirmed the application of pulsatile flow with minimum oscillations. EC responses based on morphology, nitric oxide (NO) release, metabolic activity, and cell count on day 1 and day 4 verified the presence of hemodynamic flow conditions. For the first time, it is also demonstrated that the designed bioreactor is capable of capturing EC responses to stent deployment beyond a 24-hour period with this testbed. A temporal investigation of EC responses to stent implantation from day 1 to day 4 showed significantly lower metabolic activity, EC proliferation, no significant changes to NO levels and EC's aligning locally to edges of stent struts, and random orientation in between the struts. These EC responses were indicative of stent-induced disturbances to local hemodynamics and sustained EC injury response contributing to neointimal growth and development of in-stent restenosis. This study presents a novel computationally informed 3D *in vitro* testbed to evaluate stent performance in presence of hemodynamic flow conditions found in native peripheral arteries and could help to bridge the gap between the current capabilities of 2D *in vitro* cell culture models and expensive pre-clinical *in vivo* models.

## Introduction

Peripheral artery disease (PAD) occurs due to the development of atherosclerotic plaque that results in the reduction of blood flow in arteries outside of the coronary circulation. Over 12 million people in the United States and 200 million people across the world suffer from PAD (1–3). If left untreated, it can lead to heart attack, transient ischemic attack, amputation, and renal artery disease. Even though endovascular treatments including balloon angioplasty followed by stent placement is the gold standard in revascularization of peripheral artery stenosis, it has been reported that a significant proportion of patients require secondary interventions due to complications such as in-stent restenosis and late stent thrombosis (4, 5). Clinical failure of stents not only depends on patient-specific physiological factors but also on damage caused to endothelial cells by the stent design itself, which may cause excessive harm either during implantation or in the longer term by disrupting natural blood flow patterns (6). Thus, an improved understanding of the vascular injury response caused by stent placement as a measure of stent performance is of utmost importance in increasing the patency of primary interventions.

Endothelial cells (EC) *in vivo* are constantly exposed to the combination of hemodynamic forces of shear stress, radial pressure, and circumferential stress. Hemodynamic flow conditions promote EC elongation and orientation in the direction of flow, suppress vascular smooth muscle cells (VSMC) proliferation, stimulate anti-inflammatory gene expression, and production of vasodilators and vasoconstrictors at healthy levels (7, 8). Endothelium-derived nitric oxide is a potent vasodilator, it contributes to the inhibition of VSMC proliferation creating an overall anti-thrombotic and anti-proliferative effect (9). Although stent deployment in peripheral arteries restores normal blood flow, it causes endothelial cell injury and alterations to local hemodynamics, which gives rise to complex biological processes at the cellular level, resulting in intimal hyperplasia, one of the leading causes of in-stent restenosis (10). Regions surrounded by stent struts have been characterized by poor endothelialization, impaired intercellular junctions, reduced expression of anti-thrombotic molecules, decrease in nitric oxide production, cell proliferation, and altered EC morphologies (11). Furthermore, protrusion of stent struts into the lumen results in regions of low or abnormally oscillating shear stress, which promotes inflammation, cell apoptosis, platelet activation, and inhibition of cell migration (12, 13). Thus, stent placement has a huge impact on the blood flow field developed around the stent struts and its interaction with endothelial cells in contact. Therefore, there is a need for cellular-level understanding to assess and optimize the performance of novel stent designs.

*In vivo* pre-clinical models provide a realistic platform to fully evaluate stent performance but are associated with ethical and financial concerns, with added difficulty in interpreting results from a complex biological system (14). Although *in vitro* models are unable to replicate the complexity of the vascular wall and physiological processes observed *in vivo*, they allow analysis of specific cellular components in biomimetic configurations in a controlled biochemical and biomechanical environment. Two-dimensional *in vitro* models have already provided insights into complex biological mechanisms resulting in in-stent restenosis (15, 16). Previous studies have used a parallel plate flow chamber to demonstrate the delay in endothelial repair caused by stent struts due to the formation of localized bidirectional flow which traps migrating endothelial cells (17). Wang and colleagues (18) developed a 2D *in vitro* injury model and demonstrated that endothelial restoration post stent deployment is mainly attributed to the migration of adjacent ECs with an injury scale of 2 mm and the contribution of EC adhesion to re-endothelialization increased in an injury scale-dependent way. Though 2D models prove to be sufficient for testing hypotheses, and evaluation of different materials and surface treatments but they are unable to provide a realistic 3D platform capable of delivering a combination of hemodynamics forces found *in vivo*, which are essential for endovascular device testing.

In recent years, 3D *in vitro* models have been developed to evaluate stent-cell interaction to combat clinical complications such as in-stent restenosis and thrombosis. Cardinal et al. (19) developed a 3D *in vitro* blood vessel mimic (BVM) model by subjecting human microvascular ECs seeded on polytetrafluoroethylene (ePTFE) grafts to low flow and low-pressure conditions. A cobalt-chromium bare metal stent (Guidant Corporation, Santa Clara, CA) was deployed in the BVM models. It was shown that 7 days post stent deployment, stent struts were covered by a von-Willebrand-factor-positive cell layer and optical coherence tomography (OCT) confirmed the presence of cell monolayer 14 days post stent deployment. However, a key drawback was that the BVM model consisted of a synthetic polymer scaffold limiting vessel wall compliance and was unable to provide physiological levels of tensile hoop stress essential for the analysis of biochemical responses such as the release of vasodilating and vasoconstricting agents post stent deployment. Overcoming the limitation of vessel compliance, Punchard et al. (20) investigated the impact of balloon expandable stent (Liberté^TM^ Boston Scientific Corporation, USA) on medical grade silicone tubes seeded with human umbilical vein endothelial cells (HUVECs) mimicking coronary artery biomechanical environment for 24 h. The realignment of HUVECs to local flow patterns in the presence of stent and increase in gene expression of inflammatory markers such as E-selectin, intercellular adhesion molecule-1 (ICAM-1) and vascular cell adhesion molecule-1 (VCAM-1) was observed. A similar bioreactor system was adapted for peripheral artery flow conditions by Ni Ghriallais et al. (21) to establish that a combination of self-expanding nitinol stent (Cordis S.M.A.R.T. Control nitinol stent system) deployment and vessel geometry changes (curvature) in presence of hemodynamic flow conditions resulted in cell responses associated with pro-atherosclerotic conditions and in-stent restenosis. Fibronectin-coated medical-grade silicone tubes seeded with HUVECs in straight and curved configurations were subjected to hemodynamic flow conditions for 24 h. Curved configurations showed lower NO release, higher EC numbers, and random orientation compared to straight ones. Additionally, curved stented configurations showed less viable EC's than straight configurations. Although the bioreactor systems used in previous studies have been shown to provide a combination of hemodynamic forces found *in vivo*, very few studies have verified the bioreactor design based on applied flow conditions through CFD studies. As endothelial cells are sensitive to dynamic wall shear stress patterns, it is important to evaluate bioreactor design based on applied flow conditions and compute hemodynamic quantities of interest such as time-averaged wall shear stress (*TAWSS*) and oscillatory shear index (*OSI*) which are key regulators of vascular pathophysiology (22). Furthermore, studies to date that have used silicone models have only investigated short-term cell responses (up to 24 h) post stent deployment. The rationale behind selecting a 24-h time point was that *in vivo* endothelial regrowth process begins within the first 24 h after arterial denudation. However, a short-term response of up to 24 h is unable to provide insights on EC responses contributing to neointimal growth observed at early time points (3–7 days) post stent deployment *in vivo* (23) that could help to evaluate the risk of in-stent restenosis and thrombosis at early time points.

In this study, we developed a computationally-informed perfusion bioreactor system capable of delivering physiologically relevant hemodynamic forces for peripheral artery applications. This system enabled the investigation of EC responses to stent deployment for up to 4 days in a 3D environment. The design of the bioreactor system and applied flow conditions were assessed through steady state and transient CFD analysis. The bioreactor was used to evaluate stent-cell interactions, by seeding straight compliant silicone tubes with endothelial cells, deploying a self-expanding nitinol stent, and subjecting the pseudovessel to hemodynamic flow conditions. EC response to stent deployment was assessed based on cell morphology, cell viability, cell proliferation, and nitric oxide production over acute time points of day 1 and day 4.

## Materials and Methods

### Design and Assembly of the Bioreactor System

**Figure 1:**
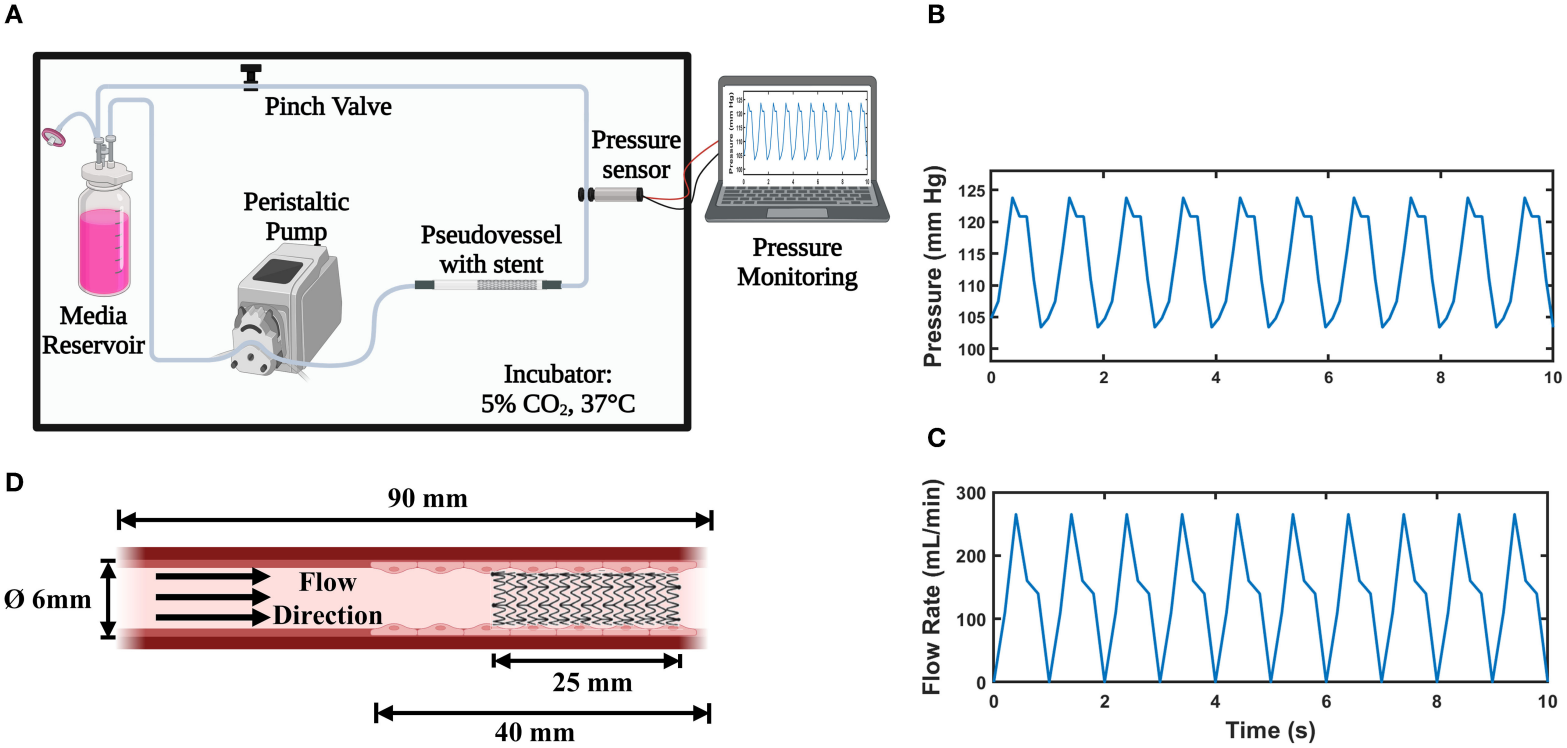
*In vitro* Bioreactor system; **(A)** Bioreactor assembly (Created with BioRender.com); **(B)** Pulsatile pressure waveform acquired from the pressure sensor; **(C)** Expected pulsatile flow profile from the peristaltic pump; **(D)** Pseudovessel comprising of HUVECs with implanted stent (Created with BioRender.com).

The bioreactor assembly shown in Figure 1A was designed to provide physiologically relevant hemodynamic conditions of flow rate, pressure, radial distention, and wall shear stress found in native peripheral arteries. It is capable of culturing multiple pseudovessels of varying geometry and sizes simultaneously. A multi-channel peristaltic pump (Masterflex L/S^®^) provides pulsatile flow at an average flow rate of 150 mL/min comparable to the flow rate found in peripheral arteries (24, 25) with a pulse frequency of 1 Hz. It pumps the cell culture media from the media reservoir to the pseudovessel through compliant platinum cured silicone tubing (inner diameter 6.4 mm, wall thickness 1.6 mm, Fisher Scientific, Ireland) forming a closed-loop system. A 0.2 μm (Fisherbrand^TM^) gas filter was attached to the media reservoir for adequate gas exchange. The entire flow loop was placed in a standard cell culture incubator for maintaining physiological levels of CO_2_ (5%), pH, temperature (37°C), and humidity (96%). The pressure in the system 80/120 mmHg was continuously monitored using a pressure sensor (UPS-HSR) attached downstream of the pseudovessel. A sampling interval of 0.1 s was specified while pressure monitoring satisfying the Nyquist sampling criterion. The mean and amplitude of the pressure waveform were regulated through the pinch valve attached downstream and basal pressure was adjusted by varying the height of the media reservoir. The pressure profile and expected flow rate curve can be seen in Figures 1B,C. A video strain extensometer measured 5% radial distention of the pseudovessel, defined as the percentage of the instantaneous outer diameter of the pseudovessel subjected to pulsatile flow to the original outer diameter of the vessel under a no flow condition.

### Verification of Bioreactor Design Through Computational Fluid Dynamics Model

**Figure 2:**
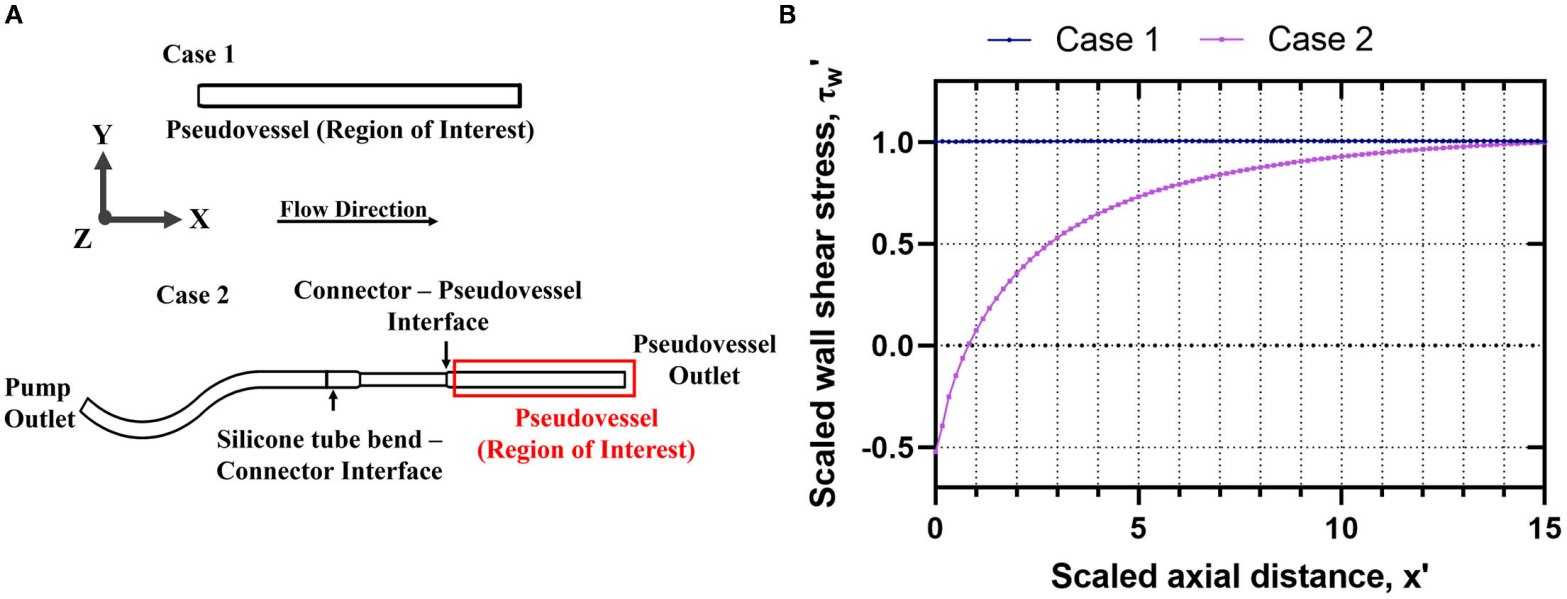
Verification of bioreactor design through steady state CFD analysis **Case 1**: Pseudovessel alone, **Case 2**: Outlet of the peristaltic pump to the pseudovessel outlet; **(A)** 2D schematic of Case 1 and Case 2 to evaluate the effects of inlet flow disturbances on the HUVECs seeded pseudovessel; **(B)** Comparison of scaled wall shear stress profile for Case 1 and Case 2.

The assembled bioreactor design and applied flow conditions were verified through steady state CFD analysis. A steady state analysis was computationally less expensive and sufficient to estimate the impact of inlet flow disturbances on the wall shear stress and velocity distribution across the pseudovessel. Once the bioreactor design was finalized, a transient CFD analysis was performed to estimate hemodynamic quantities of interest such as *TAWSS* and *OSI*. A 3D geometric model of the bioreactor assembly from the outlet of the peristaltic pump to the pseudovessel outlet was created using Autodesk Inventor^®^ 2021. The model was imported to OpenFOAM HELYX v.3.3.2, where steady state flow simulations were performed. The impact of inlet disturbances introduced by both a bend in the silicone tubing and a connector upstream of the pseudovessel were assessed. The bend in the silicone tubing had to be considered due to the space limitation of the bioreactor system within the dimensions of the incubator. The experimental setup from the outlet of the peristaltic pump to the pseudovessel outlet (Case 2) as the computational domain was compared with the pseudovessel alone (Case 1) as the computational domain as shown in Figure 2A. The mesh was refined at the interface of silicone tubing-connector (converging section) and connector-pseudovessel (sudden expansion), as seen in Figure 2A, to accommodate for high-velocity gradients, adverse pressure gradients, and flow separation and reattachment. Additionally, five layers of prismatic cells with a growth factor of 1.25 and a final layer thickness ratio of 0.4 was specified to resolve gradients near the wall. A grid convergence study was conducted in accordance with the guidelines from Examining spatial grid convergence (26) using Richardson extrapolation and Grid Convergence Index (GCI) to estimate the discretization error. The total number of cells for Case 1 and Case 2 were 48,960 million and 210,000 million, respectively. A convergence criterion of 10^−5^ was specified for both pressure and velocity residuals. Additional information on mesh generation and mesh convergence study can be found in the Supplementary Material (Section Introduction). A rigid wall no-slip boundary condition was specified for the pseudovessel wall, zero pressure at the domain outlet, and a fully developed Hagen-Poiseuille velocity profile to the inlet, where a mean velocity of 0.08 m/s (equivalent to an average flow rate of 150 ml/min) was defined. The cell culture medium behaves as a Newtonian fluid of density (ρ = 1,000 kg/m^3^ and dynamic viscosity μ = 7.8 × 10^−4^ Pa.s).

In order to compare the results of the simulated cases and translate the *in vitro* results more easily to an *in vivo* situation, where rheological conditions differ, the physical quantities of the simulations were scaled. Velocity field, axial and radial position, and wall shear stress were converted into dimensionless forms using Eq (1)


(1) $$\begin{array}{l} \begin{array}{c} \begin{array}{l} x^{\prime }=\frac{x}{D}\;r^{\prime }=\frac{r}{D}\;u^{\prime }\frac{u}{u_{mean}}\;\\ \tau_{w}{}^{\prime }=\frac{\tau_{w}}{\tau_{w_{mean}}}\;\tau_{w_{mean}}=\frac{32\mu Q}{\pi D^{3}} \end{array} \end{array} \end{array}$$

where *D* is the diameter of the pseudovessel, *x* is axial position, *r* is the radial position, *u* is the velocity field, τ_*w*_ is the wall shear stress, *u*_*mean*_ and τ_*w*_*mean*__ represent the average velocity and average wall shear calculated from Hagen–Poiseuille flow. The three dimensionless numbers that characterized flow behavior in this study were Reynolds number *Re* =680, the Dean number *De* =*95* and the Womersley number *Wo* =*13*. To simulate pulsatile flow conditions in the pseudovessel, a pulsatile flow waveform shown in Figure 1C, was imposed at the inlet. Simulations were performed over three pulsatile cycles and results were analyzed for the third pulse of the simulation to remove flow-dependent effects. Time-averaged wall shear stress (*TAWSS*) and Oscillatory shear index (*OSI*) distributions were computed as


(2) $$\begin{array}{l} \begin{array}{c} TAWSS=\frac{1}{T}\int_{0}^{T}|\tau_{w}|\;dt \end{array} \end{array}$$

(3) $$\begin{array}{l} \begin{array}{c} OSI=\;\frac{1}{2}\left(1-\frac{\left|\int_{0}^{T}\tau_{w}dt\right|}{\int_{0}^{T}|\tau_{w}|\;dt}\right) \end{array} \end{array}$$

where *T* is the period of a pulse. The wall shear stress was incrementally integrated using data obtained from all time steps. The *OSI* varies between 0 and 0.5 with a value of 0 observed in regions of unidirectional flow and a value of 0.5 observed in regions of fully oscillatory flow (27). All 3-D plots were generated in Paraview v.5.9.1.

### Cell Culture

HUVECs (PromoCell) were thawed in a T-25 flask at a recommended seeding density of 10,000 cells/cm^2^ and cultured in endothelial cell growth medium (EGM^TM^-2, CC-3162) supplemented with 5% fetal bovine serum (FBS), 1% penicillin-streptomycin (P/S). At 80–90% of confluency, the cells were trypsinized and passaged in T-75 flasks with a sub-cultivation ratio of 1:3. The endothelial cell growth medium was replenished every 48 h and cells were maintained with 5% CO_2_ at 37°C and 96% humidity.

### Pseudovessel Formation

Medical grade silicone tubes (RT-601, Elastosil, Wacker) with an inner diameter of 6 mm, an outer diameter of 7 mm, length of 90 mm, wall thickness of 1 mm, and an elastic modulus of 0.5 MPa, and tensile strength of 6 MPa were used to fabricate pseudovessels shown in Figure 1D. The silicone tubes were autoclave sterilized, coated with 8 μg mL^−1^ fibronectin solution (Sigma) for 6–8 h in the incubator, and rinsed with Phosphate Buffer Saline solution (PBS, Sigma) to remove excess fibronectin solution. HUVECs between passage 4 and 6 were seeded at a density of 130,000 cells/cm^2^ (28, 29) and cultured for 48 h under slow rotation with media change performed after 24 h of seeding. After 48 h (day 0 of the experiment), a small portion (length = 1 cm) of the pseudovessel was removed to check the confluency. Unstented and stented pseudovessels subjected to hemodynamic flow conditions were compared to unstented and stented pseudovessel in static conditions as controls (*n* = 3 pseudovessels per experimental group) over 1- and 4-day time points.

### Stent Deployment

A commercial self-expanding nitinol stent (7 × 40, Zilver^®^ Flex 35 Biliary Vascular Stent, Cook Medical, Bloomington, Indiana, U.S.A) with a strut width of 105 μm and strut thickness of 197 μm was used for the study. The nitinol stent was crimped from a nominal diameter of 7 to 2 mm inside glass tubes (inner diameter 2 mm, outer diameter 4 mm, and length 150 mm) using a radial compression station (Blockwise Model RVS) similar to previous studies conducted (30–32). The glass tube with the stent was ethanol sterilized followed by drying in sterile flowhood for 2–3 h and rinsing with PBS to remove any traces of ethanol. The nitinol stent was deployed inside the cell seeded pseudovessel by pushing the stent gently with a sterile probe and removing the glass tube inside the flowhood, similar to the unsheathing process. Additionally, steady state CFD analysis was used to determine the scaled axial distance at which the stent was deployed in the pseudovessel to minimize the effect of inlet flow disturbances on HUVECs. It was ensured that the glass tube did not come in contact with the endothelial cells seeded on the pseudovessel wall and no damage to the endothelium was caused by the glass tube during the deployment process. The stent covered 62.5% of the cell seeded area of the pseudovessel as shown in Figure 1D.

### Media Perfusion

Pseudovessels (*n* = 3 per experimental group) were subjected to hemodynamic flow conditions for 1- and 4-days. Cell culture media was renewed every 48 h and the pH of the media was monitored regularly. At the start-up of the flow experiments, a mean pulsatile flow rate of 60 ml/min was applied to provide media circulation to the HUVECs monolayer with minimal shear. This was followed by gradually ramping up the flow rate to 150 ml/min after 4–6 h of pre-conditioning to minimize any damage to the HUVECs monolayer. This flow rate was maintained continuously over the next 4 days.

### Cell Morphology and Cell Number

At each time point, HUVECs in the pseudovessels were washed in PBS (Sigma) and fixed in 10% formalin (Sigma) for 15 min. The fixed pseudovessels were first dipped in hemotoxylin (Sigma) solution for 5 min followed by a bath in 1% eosin solution (w/v in distilled water) for 3 min and washed with deionized water to remove the excess stain as previously described (33). Furthermore, pseudovessels were sectioned longitudinally with a scalpel, mounted between two histological slides followed by imaging under a brightfield microscope (BX43 Olympus^®^), and analyzed in cellSens [Ver.1.14] software. Cell orientation and cell count were quantified in ImageJ software by adaptive thresholding. A particle analysis tool was used to measure the orientation of each cell with best-fit ellipses (20, 21). It should be noted that for the purpose of this study, only the cells orienting in the direction of flow (0–30° orientation angle) were considered for analysis. Cell number was determined by counting cells in 5 representative sections for each pseudovessel.

### Cell Metabolic Activity

Alamar blue was used to assess cellular metabolic activity based on mitochondrial reduction. After each time point, pseudovessels were removed from the bioreactor, rinsed with PBS followed by the addition of 10 % (v/v) solution of Alamar Blue^TM^ in culture media, and rotated for 4 h in the incubator protected from light as previously described (34). Fluorescence of the cell conditioned alamar blue was measured at the excitation wavelength of 560 nm and emission wavelength of 590 nm with a microplate reader (Bio Tek Gen5, Synergy).

### Nitric Oxide Production

Nitric oxide levels were quantified using nitric oxide fluorometric assay kit (482655, Sigma-Aldrich). All reagents, solutions, and the standard curve were prepared in accordance with manufacturers protocol. At each time point, pseudovessels were removed from the bioreactor. The pseudovessels were rinsed with PBS followed by adding culture media and rotated for 2 h in the incubator. 20 μl of the cell-conditioned media was added to each well of the 96 well plate and fluorescence was measured at the excitation wavelength of 360 nm and emission wavelength of 430 nm (Bio Tek Gen5 Microplate reader).

### Statistical Analysis

All results presented in this study were representative of three independent experiments. The data were presented as mean ± standard deviation. A two-way analysis of variance (ANOVA) followed by *post-hoc* Tukey test was performed in GraphPad Prism. For the purpose of this study, flow groups were compared to their static counterparts (static unstented vs. flow unstented, static stented vs. flow stented) to assess EC response to flow and no flow conditions based on EC metabolic activity, NO production, EC count and EC orientation for day 1 and day 4 time points. Similarly, a statistical comparison was performed between unstented groups and their corresponding stented groups (static unstented vs. static stented, flow unstented vs. flow stented) to account for implications of stent deployment on EC response for day 1 and day 4 time points. A time-course analysis of EC response was carried out for all experimental groups from day 1 to day 4 time points (static unstented day 1 vs. static unstented day 4, static stented day 1 vs. static stented day 4, flow unstented day 1 vs. flow stented day 4, flow stented day 1 vs. flow stented day 4). A p^**^ <0.01 was considered as highly statistically significant, p^*^ < 0.05 was considered statistically significant.

## Results

### Numerical Verification of the Bioreactor Design

**Figure 3:**
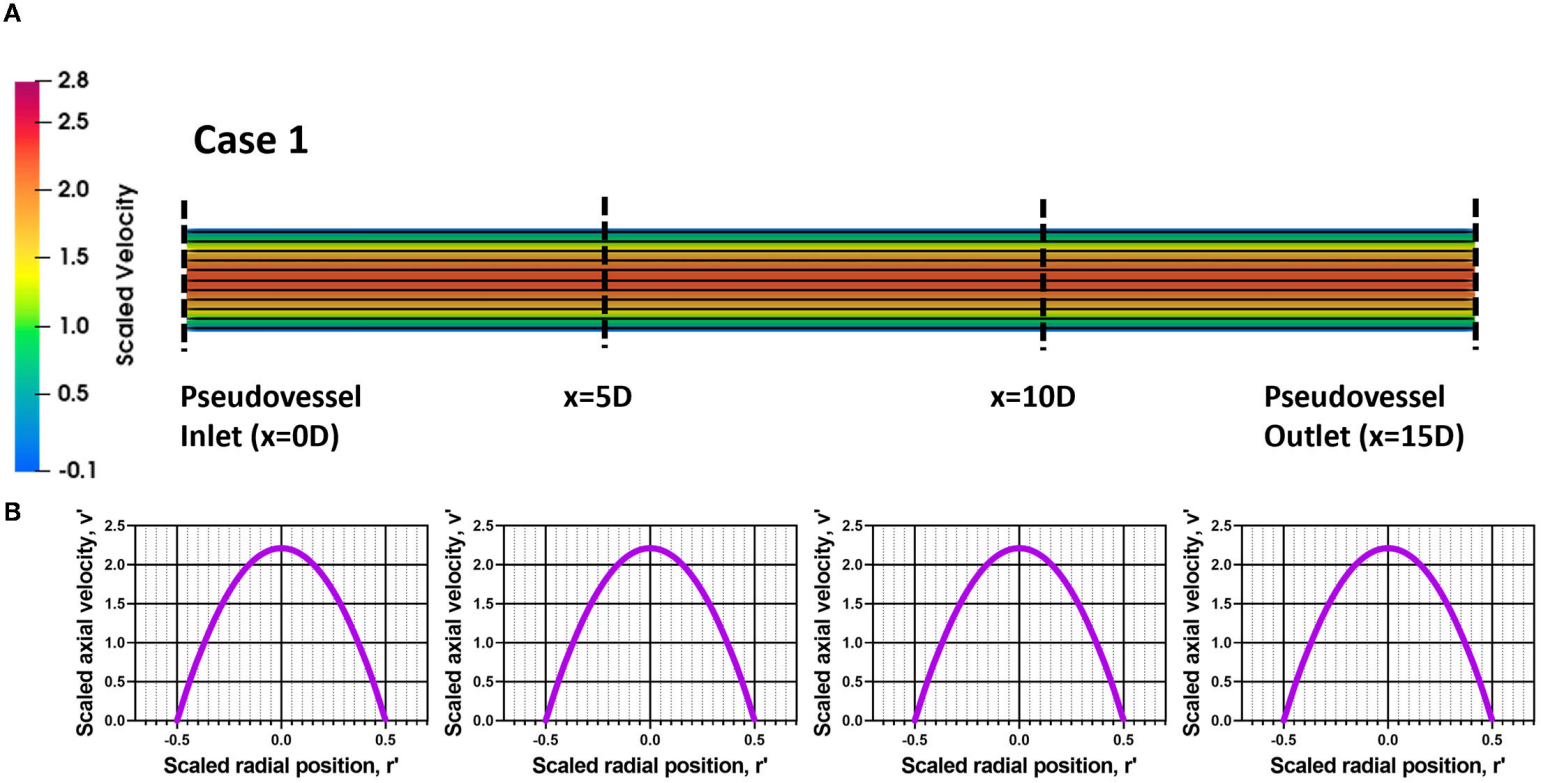
Assessment of flow profile for Case 1; **(A)** Scaled in-plane velocity distribution of longitudinal slice with velocity streamlines; **(B)** Scaled axial velocity vs. scaled radial position plot along the axial length for Case 1.

**Figure 4:**
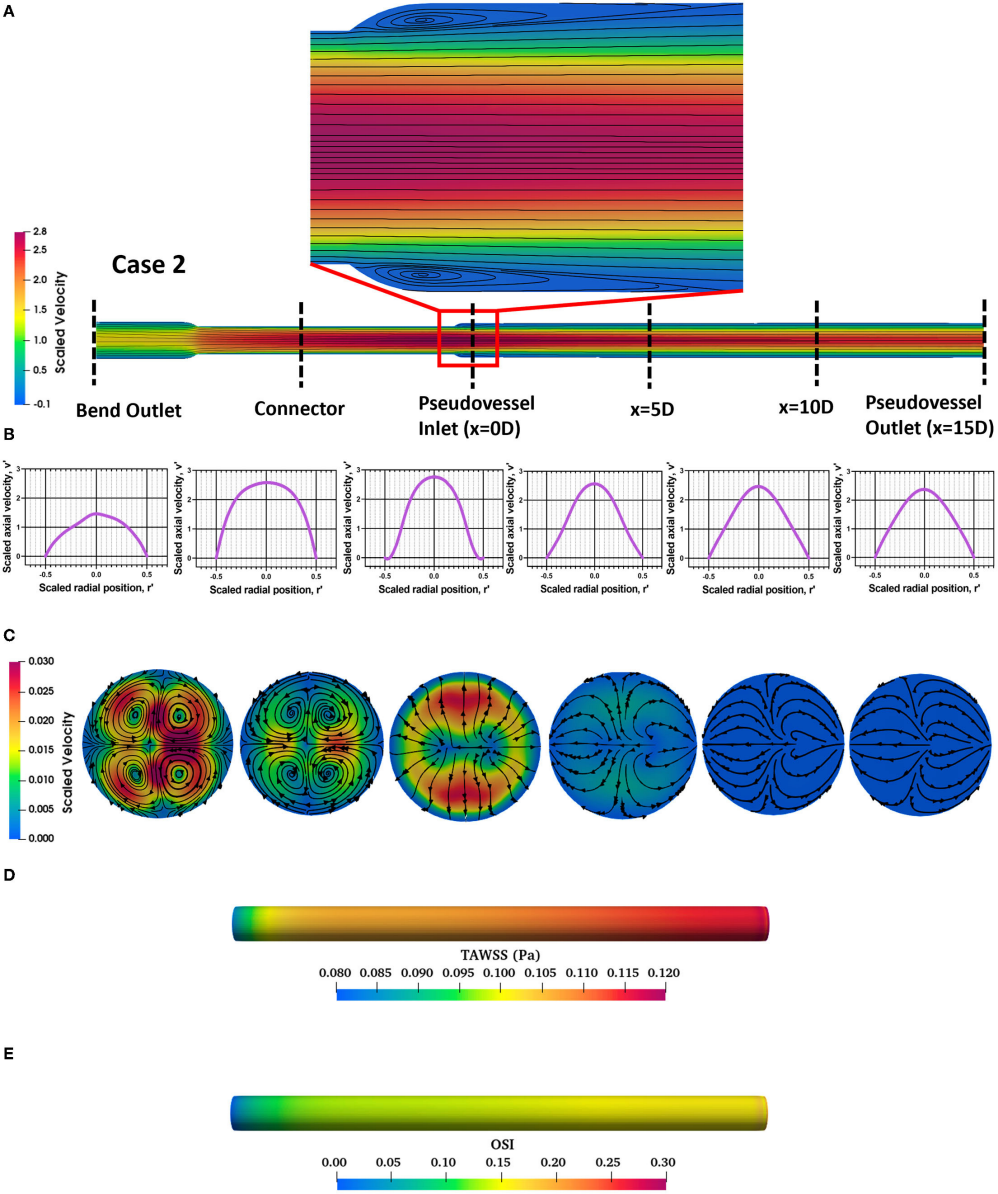
Assessment of flow profile for Case 2; **(A)** Scaled in-plane velocity distribution of longitudinal slice with velocity streamlines showing formation of recirculation zones at the edges; **(B)** Scaled axial velocity vs. scaled radial position plot along the axial length for Case 2; **(C)** Cross-sectional slices of scaled in-plane velocity distributions along the axial length for Case 2; **(D)** Time-averaged wall shear stress (*TAWSS*) distribution across the pseudovessel; **(E)** Oscillatory shear index (*OSI*) distribution across the pseudovessel.

The steady state CFD analysis for Case 2 (pseudovessel with inlet flow disturbances) resulted in the formation of a pair of secondary Dean vortices introduced by the bend in silicone tubing and recirculation zones at the edges of the connector and pseudovessel interface, as shown in **Figures 4A,C** compared to uniform fully developed velocity profile for Case 1 in Figures 3A,B. However, we observe that Dean vortices and recirculation zones gradually start disappearing further downstream from the pseudovessel inlet, as can be seen from the cross-sectional scaled in-plane velocity distributions and axial velocity profile in Figures 4B,C. To estimate the influence of inlet disturbances on the wall shear stress along the pseudovessel, wall shear stress for both cases were compared. It was observed that at an axial distance equivalent to 10.8 times the diameter from the pseudovessel inlet, the error in wall shear stress between Case 2 and Case 1 was reduced to 5% (acceptable range), as shown in Figure 2B. Thus, it served as a design criterion for the distance at which stent was deployed in HUVECs seeded pseudovessel and ensured minimal effect of inlet disturbances on the endothelial cells. *TAWSS* and *OSI* distributions for the pseudovessel from transient CFD simulations are shown in Figures 4D,E. We report a *TAWSS* in the range 0.1–0.12 Pa and an *OSI* < 0.2 downstream of the pseudovessel (10.8 times the diameter from the pseudovessel inlet) where the stent was deployed.

### Cell Metabolic Activity

**Figure 5:**
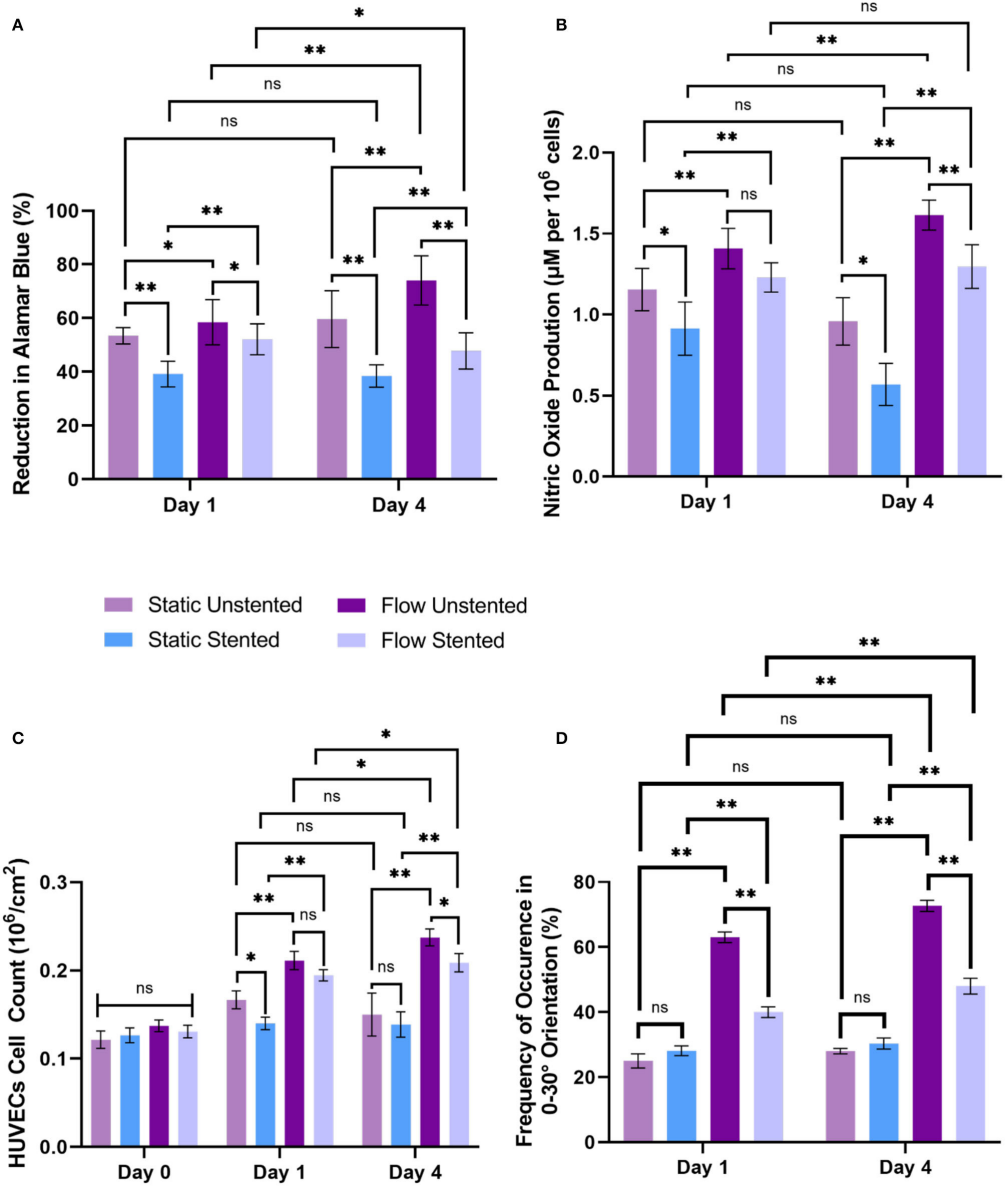
Biochemical response of EC, cell count, and cell orientation to hemodynamic flow conditions and stent deployment at day 1 and day 4, **(A)** cell metabolic activity, **(B)** nitric oxide release, **(C)** cell count observed from Hemotoxylin and Eosin (H&E) images, and **(D)** cell orientation in the range 0-30° orientation in the direction of flow was assessed for all experimental groups. Error bar represents mean ± S.D, ^*^ represents *p* < 0.05, ^**^ represents *p* < 0.01 and ns being non-significant.

Comparing EC metabolic activity between flow groups to their corresponding static groups showed a significant increase in metabolic activity for the flow unstented group compared to the static unstented (*p* < 0.05) group while a highly significant increase was found for the flow stented group compared to static stented (*p* < 0.01) on day 1. The day 4 time point revealed a highly significant increase (*p* < 0.01) between all flow groups compared to their corresponding static groups as shown in Figure 5A. A significant decrease (*p* < 0.05) in cellular metabolic activity was seen for all stented groups compared to their unstented counterparts irrespective of flow conditions for both day 1 and day 4 time points. Temporal analysis of EC metabolic activity revealed a highly significant increase (*p* < 0.01) in metabolic activity for the flow unstented group from day 1 to day 4 (58.49 ± 8.42 % on day 1 vs. 74.04 ± 9.16 % at day 4). In contrast to this, a significant decrease (*p* < 0.05) in metabolic activity was found for stented groups under flow conditions between day 1 and day 4 (52.11 ± 5.76 % at day 1 vs. 47.80 ± 6.73 % at day 4). For static groups, no significant difference in metabolic activity was observed from day 1 to day 4 time point.

### Nitric Oxide Production

Nitric oxide results are shown in Figure 5B revealed a highly significant increase (*p* < 0.01) in NO release for all flow groups compared to their static counterparts at day 1 and day 4. Stent deployment resulted in a significant decrease (*p* < 0.05) in NO production in static stented groups compared to static unstented groups on both day 1 and day 4. The addition of flow to the stented group showed no significant decrease in NO release at day 1 compared to the flow unstented group. However, a highly significant decrease was found between the flow unstented and flow stented group at day 4 time point (*p* < 0.01). A quantitative comparison of NO levels from day 1 to day 4 showed a highly significant increase in NO production for the flow unstented group on day 4 compared to day 1 (*p* < 0.05, 1.41 ± 0.06 μM per 10^6^ cells at day 1 vs. 1.61 ± 0.05 μM per 10^6^ cells at day 4). Moreover, no statistical differences in NO release were observed for flow stented, static unstented, and static stented groups from day 1 to day 4.

### Cell Number

Statistical analysis of EC count based on histology images on day 0 time point showed no significant difference in EC count for all experimental groups as shown in Figure 5C. A highly significant increase in HUVECs number (*p* < 0.01) was measured for all flow experimental groups compared to their corresponding static controls for both day 1 and day 4 time points. Stent implantation in pseudovessels resulted in significant decrease in EC count (*p* < 0.05) in static stented group than static unstented group on day 1 time point but no significant difference in EC count was observed for day 4 time point. Interestingly, no statistical difference in EC count was seen for flow stented group compared to flow unstented group on day 1 but a significant difference was observed for day 4 time point. A time-course analysis of EC count from day 1 to day 4 time point showed a significant increase in EC number in flow unstented group (*p* < 0.01, 0.21 ± 0.01 10^6^ cells/cm^2^ at day 1 vs. 0.237 ± 0.004 10^6^ cells/cm^2^ at day 4) and flow stented group (*p* < 0.01, 0.20 ± 0.006 10^6^ cells/cm^2^ at day 1 vs. 0.20 ± 0.004 10^6^ cells/cm^2^ at day 4). However, no statistical difference in EC number was seen for static unstented and static stented groups from day 1 to day 4 time point.

### Cell Morphology and Orientation

**Figure 6:**
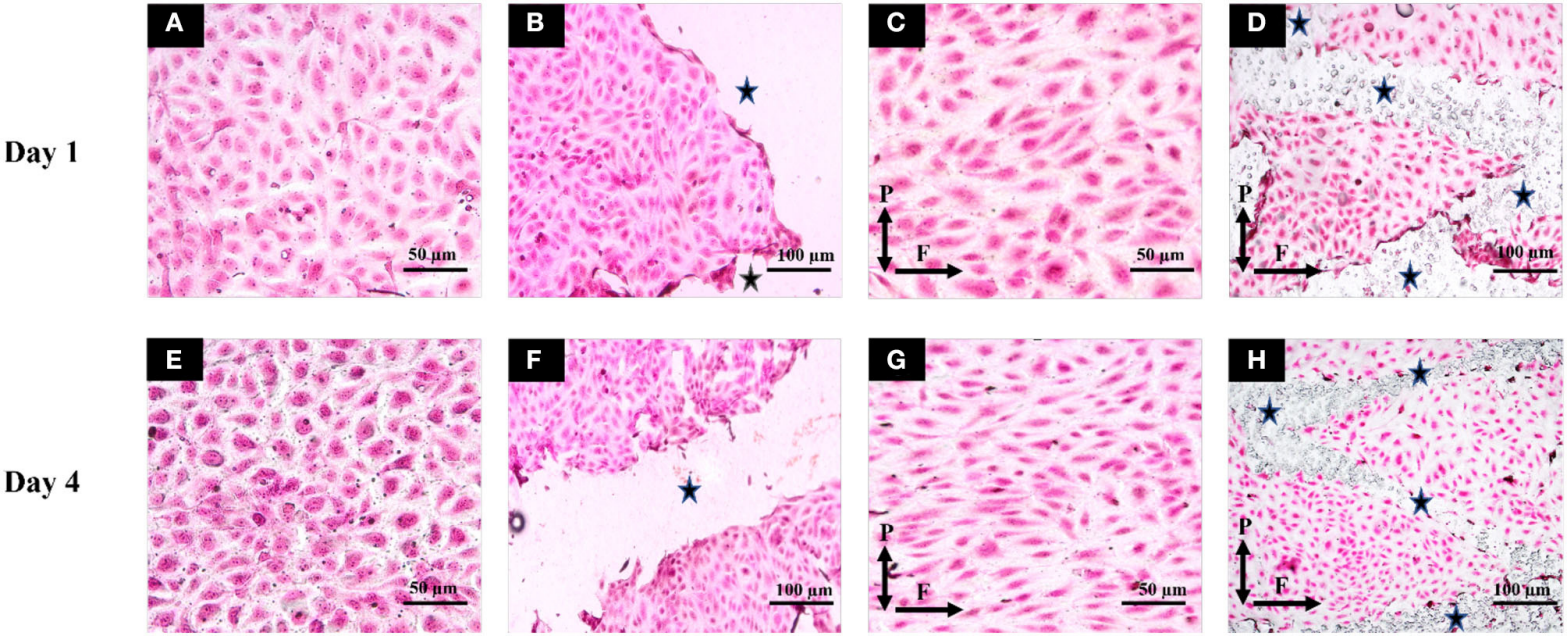
Hematoxylin and Eosin staining was performed and images of flow and static groups were taken for Day1 (top row) and Day 4 (bottom row). **(A)** Static unstented for Day 1; **(B)** Static stented for Day 1; **(C)** Flow unstented for Day 1; **(D)** Flow stented for Day 1; **(E)** Static unstented for Day 4; **(F)** Static stented for Day 4; **(G)** Flow unstented for Day 4; **(H)** Flow stented for Day 4. Scale bars represent 50 μm **(A,C,E,G)** and 100 μm **(B,F,D,H)**. P and F correspond to the direction of forces (pulsatile pressure and flow). * represents stented regions in the pseudovessel.

All static groups showed a complete random orientation and a cobblestone morphology on both day 1 and day 4 time points as shown in Figures 6A,E. In contrast to this, the majority of EC's showed elongated and spindle like morphology in flow unstented group (Figures 6C,G) while a combination of spindle like and cobblestone morphologies was observed for flow stented groups (Figures 6D,H). Cell orientation results (Figure 5D) revealed that highly significant percentage of EC's aligned in 0–30° orientation in direction of flow in flow unstented groups (*p* < 0.01) compared to flow stented, static unstented and static stented groups on day 1. A highly significant increase in EC alignment to the direction of flow (*p* < 0.01, 63 ± 1.65% at day 1 vs. 72.67 ± 1.7% at day 4) was observed for flow unstented group from day 1 to day 4.The alignment of the cells in direction of flow was significantly increased in flow stented group from day 1 to day 4 (*p* < 0.01, 40.3 ± 2.1% at day 1*vs*. 48.5 ± 2.4% at day 4), but there was a complete random orientation observed for the static counterpart at both time-points. It can be seen in Figures 6D,H, EC aligning local to the stent struts with more cell alignment near strut edges and random orientation in the central region in between the stent struts. A time-based analysis of EC orientation showed no significant change in the percentage of EC alignment in static groups from day 1 to day 4.

## Discussion

This study demonstrated, for the first time, the development of a computationally-informed bioreactor system that can capture EC response to stent deployment in presence of hemodynamic flow conditions comparable to native peripheral arteries for up to 4 days. Our study is the first to assess the impact of flow disturbances introduced by connections upstream of cell seeded vascular constructs in a bioreactor system and investigate cellular response on such a platform beyond a 24-h period. EC response based on cell metabolic activity, cell count, NO production, and morphology was captured for unstented and stented pseudovessels subjected to static and hemodynamic flow conditions for acute time points of day 1 and day 4. Our results from day 1 verified that the designed bioreactor system can deliver a combination of hemodynamic forces found *in vivo* and is able to capture expected EC responses to stent deployment. Stent implantation in EC seeded silicone pseudovessels resulted in a significant increase in EC proliferation, lower metabolic activity, no significant changes in NO levels, and ECs aligning local to stent struts with random orientation in between stent struts from day 1 to day 4. A time course analysis of EC injury response suggested that stent placement leads to regions of disturbed shear stress inducing EC proliferation and showed biochemical responses of a dysfunctional endothelium not restored to its normal function. These responses contribute to neointimal growth and increase the risk of development of in-stent restenosis. In this study, we also highlighted the importance of evaluating the impact of inlet flow disturbances introduced by connections upstream of cell seeded vascular constructs on the wall shear stress through a detailed CFD analysis. Our results demonstrated that at a dimensionless axial distance of 10.8 times the diameter from the pseudovessel inlet, the impact of inlet flow disturbances such as Dean vortices and recirculation zones was minimal and was used as a design criterion for stent deployment in EC seeded pseudovessels. Transient CFD analysis demonstrated that EC seeded regions of the pseudovessel were exposed to a *TAWSS* between 0.1 and 0.12 Pa and an *OSI* value <0.2 confirming the application of pulsatile flow with minimum oscillations. This computationally informed *in vitro* bioreactor provides a robust platform to study the short-term stent-artery interactions, helping to bridge the gap between the current capabilities of 2D *in vitro* cell culture models and costly pre-clinical *in vivo* models.

After subjecting pseudovessels under hemodynamic flow conditions for day 1, a significant increase in EC metabolic activity, EC count, and NO release was observed for flow experimental groups compared to their corresponding static groups as shown in Figures 5A–C. Our histology images showed a significantly higher percentage of ECs exposed to pulsatile shear stress showing elongated spindle like morphology (Figures 6C,D) and aligned in 0–30° orientation in the direction of flow (Figure 5D) compared to their corresponding static counterparts where ECs were characterized by cobblestone morphology and random orientation at day 1. As a result, EC responses at day 1 verify the presence of hemodynamic flow conditions delivered by the designed bioreactor system consistent with previous *in vitro* findings (21, 35, 36). Furthermore, stent implantation in pseudovessels resulted in significantly lower metabolic activity, NO production, and EC number as opposed to their corresponding unstented groups under static conditions, which is in agreement with previous findings where stent deployment leads to severe endothelial injury and dysfunction resulting in reduced EC number, metabolic activity, and NO release at day 1 time point (37–39). Interestingly, the application of flow to the stented groups resulted in significantly lower metabolic activity compared to unstented groups, however, no significant decrease was observed for NO release and EC number at day 1. This can be attributed to minimal shearing off of ECs in stented pseudovessels in presence of stable hemodynamic flow conditions with minimal inlet flow disturbances and highlights the importance of design verification of bioreactor systems through CFD studies. Thus, EC responses at day 1 confirmed that the designed bioreactor system can deliver a combination of hemodynamic forces and is able to capture expected EC responses to stent deployment.

Although the day 1 time point provided important insights on EC responses to hemodynamic flow conditions immediately post stent implantation, the assessment period was too short to elucidate the potential role played by ECs in pathological processes such as neointimal growth and development of in-stent restenosis beyond this timeframe. For the first time, we report a significantly lower EC count, metabolic activity, and NO release in flow-stented groups compared to flow unstented groups at the day 4 time point (Figures 5A–C). These results were similar to the trends observed on day 1, owing to sustained EC injury response and changes to hemodynamic flow conditions induced by stent deployment. This would result in decreased eNOS expression leading to increased VSMC proliferation, aggregation of platelets, and deposition of extracellular matrix proteins and leukocytes, contributing to neointimal growth and development of in-stent restenosis (40, 41). Temporal analysis of EC responses from day 1 to day 4 in flow unstented groups showed a significant increase in NO release (Figure 5B) confirming the presence of an antiproliferative and antithrombotic environment representative of the physiological environment found *in vivo* (9, 42). Prolonged exposure of stented groups to hemodynamic flow conditions from day 1 to day 4 indicated a significant increase in EC proliferation (Figure 5C) as stent placement could have induced local flow disturbances, triggering a response that would be inflammatory and thrombogenic. Our observations are in support of *in vivo* studies where early neointimal growth and complete endothelization of the neointima at day 7 were reported in murine and porcine animal models (43, 44). Despite an increase in cell proliferation in the stented groups on prolonged exposure to shear stress, no statistical difference was found between NO levels from day 1 to day 4 (Figure 5B). This could be due to the development of regions of disturbed shear stress and an injured endothelium that is not fully restored to its normal function due to stent implantation, consistent with *in vivo* events (45, 46). Stent placement in pseudovessels resulted in a highly significant percentage of cells aligning in 0–30° orientation from day 1 to day 4 time point as shown in Figure 5D. Additionally, this alignment was more pronounced at the edges of the stent struts, shown in Figure 6H, while a more random orientation was observed toward the center in between stent struts characterized by a combination of elongated spindle like and cobblestone EC morphology in support of *in vivo* observations (47, 48). A possible explanation for this altered EC morphology comes from previous studies where it has been shown that stent struts induce areas of downstream flow reversal and formation of recirculation zones resulting in the random orientation of EC's while accelerated flow over the edges of the stent struts results in high shear rates initiating platelet aggregation and activation contributing to neointimal growth (11, 49). Therefore, temporal analysis of EC response to a combination of hemodynamic forces and stent placement based on cell proliferation, metabolic activity, NO production, and morphology provides valuable insights into the contribution of endothelial cells in the arterial healing process and neointimal growth at early time points *in vivo*. This would enable the development of novel therapies and stent designs that promote re-endothelialization of stented segments and restoration of ECs to their normal functionality at acute time points reducing the risk of in-stent restenosis.

The developed bioreactor system and study present some limitations. In this study, ECs are exposed to time-averaged wall shear stress of 0.1–0.12 Pa compared to a range of 0.3–1.3 Pa found in peripheral arteries of healthy human subjects at rest (50). This is due to the viscosity mismatch between cell culture media (7.8 × 10^−4^ Pa.s) and blood viscosity (3.5 × 10^−3^ Pa.s) which had an important effect on EC response in this study. Temporal analysis of EC proliferation in the flow unstented group from day 1 to day 4 time point (Figure 5C) showed a significant increase in EC proliferation. This proliferative EC response in flow unstented group where physiological flow conditions persist *in vivo* is likely due to exposure of ECs to regions of low shear stress. Low wall shear stress has been associated with increased EC proliferation, irregular EC morphologies, reduced NO production, and cell apoptosis leading to pathological conditions (12, 13, 51). This could be overcome in the future by the inclusion of additives such as dextran in the cell culture media to match blood viscosity (52). However, it would be important to first demonstrate that such agents would have minimum effect on endothelial cell response. A straight healthy artery model has been used for verification of the bioreactor system in this study. It is well known that stents are not deployed in healthy artery models but the aim of such a model is to provide insights into the effects of varying design parameters with applicability to a range of clinical situations. The significant challenges of maintaining a long-term cultural environment meant that the temporal response of HUVECs to a combination of stenting and shear stress was still limited to 4 days, which provided insight into acute responses to shear stress. Further development of the bioreactor system would seek to extend this timeframe to assess cell responses to chronic shear stress to study disease progression and processes that occur in later stages post stent deployments, such as stent thrombosis and late stent restenosis. In the future, a stented pseudovessel would be included in the CFD analysis of the designed bioreactor system to assess the impact of stent deployment on the hemodynamic metrics. A major advantage of the bioreactor system developed here is that it is highly adaptable to mechanobiological analysis, secretome analysis, and other cell types. Future studies may include the addition of VSMC, circulating endothelial progenitor, and blood cells to evaluate the performance of varied stent designs and the risk of in-stent restenosis and late stent thrombosis. Additionally, the designed bioreactor could accommodate more complex arterial geometries such as 3D printed patients specific and bifurcation models.

## Conclusion

In summary, a computationally-informed bioreactor system has been designed capable of delivering a combination of hemodynamic forces found in native peripheral arteries for endovascular stent device testing. The design of the bioreactor system and applied flow conditions were verified by steady state and transient CFD models. *TAWSS* in the range 0.1–0.12 Pa distal from the pseudovessel inlet (10.8 diameter distance) and an *OSI* <0.2 resulted in pulsatile flow with minimum oscillations. As a proof of concept, it is demonstrated that the designed bioreactor system can capture the deployment of a self-expanding nitinol stent and its interaction with a monolayer of endothelial cells subjected to hemodynamic flow conditions comparable to native peripheral arteries for up to 4 days. EC response from day 1 to day 4 in stented groups resulted in increased EC proliferation, lower metabolic activity, no significant changes in NO levels with EC's aligning local to edges of the stent struts, and random orientation in between the struts owing to sustained EC injury and changes to local hemodynamic flow conditions induced by stent implantation. These EC injury responses provide a better understanding of the role played by endothelial cells in the vascular healing process and neointimal growth post stent deployment at early time points. The developed bioreactor platform will be beneficial in designing novel stent designs and strategies to promote re-endothelialization of stented segments and minimize the risk of in-stent restenosis.

## Data Availability Statement

The raw data supporting the conclusions of this article will be made available by the authors, without undue reservation.

## Ethics Statement

Ethical review and approval was not required for this study in accordance with the local legislation and institutional requirements.

## Author Contributions

ED, TV, RH, and CD: conceptualization and supervision. SN, JS-T, RH, ED, and TV: methods and investigation. SN: original draft preparation and visualization. ED, TV, CD, RH, and JS-T: review and editing. All authors contributed to the article and approved the submitted version.

## Funding

This project has received funding from the European Union's Horizon 2020 research and innovation program under Marie Skłodowska Curie Grant Agreement No. 813869. This publication reflects only the author's view, and the REA is not responsible for any use that may be made of the information it contains.

## Author Disclaimer

This publication reflects only the author's view, and the REA is not responsible for any use that may be made of the information it contains.

## Conflict of Interest

SN is on her secondment at Vascular Flow Technologies Dundee, United Kingdom. RH and CD are employed by Vascular Flow Technologies Dundee, United Kingdom. The remaining authors declare that the research was conducted in the absence of any commercial or financial relationships that could be construed as a potential conflict of interest.

## Publisher's Note

All claims expressed in this article are solely those of the authors and do not necessarily represent those of their affiliated organizations, or those of the publisher, the editors and the reviewers. Any product that may be evaluated in this article, or claim that may be made by its manufacturer, is not guaranteed or endorsed by the publisher.
