# Supplementary material for: Design and Verification of a Novel Perfusion Bioreactor to Evaluate the Performance of a Self-Expanding Stent for Peripheral Artery Applications
Source: Front Med Technol. 2022 Jun 21;4:886458. doi: 10.3389/fmedt.2022.886458 (PMC9253816; doi:10.3389/fmedt.2022.886458)
Supplement: Supplementary file 1 [file Data_Sheet_1.docx]

Design and Verification of a Novel Perfusion Bioreactor to Evaluate the Performance of a Self-expanding Stent for Peripheral Artery Applications

Swati Nandan^1,2^, Jessica Schiavi-Tritz^1^, Rudolf Hellmuth^2^, Craig Dunlop^2^, Ted J. Vaughan^1*^, Eimear B. Dolan^1*^

^1^Biomedical Engineering and Biomechanics Research Centre (BioMEC), School of Engineering, College of Science and Engineering, National University of Ireland Galway.

^2^Vascular Flow Technology, Dundee, United Kingdom

Supplementary Material

# Mesh Generation and Mesh Convergence Study

The computational mesh for both cases were generated using OpenFOAM HELYX v.3.3.2. Mesh convergence study was performed to assess the accuracy of the simulations for a given mesh grade. Three mesh grades were considered for the mesh convergence study. For the purpose of this study, the wall shear stress distribution was of particular importance and therefore wall shear stress results (quantity of interest) were compared for each mesh grade. The details of the mesh convergence study for Case 1 and Case 2 conducted in accordance with the guidelines from Examining spatial grid convergence to estimate discretization error [1] has been shown below.

**Case 1:** Pseudovessel alone

The details of the mesh generated for Case 1 has been shown in Supplementary Material Table 1 and Supplementary Material Figure 1. The plot shown in Supplementary Material Figure 2 demonstrates that as the mesh is refined further, the wall shear stress values approaches asymptotic range of convergence with Richardson extrapolation value obtained at zero cell size. In this study, Grid Convergence Index (GCI) was also evaluated to verify if the study was conducted with mesh grade sufficiently fine for asymptotic mesh convergence. GCI is defined as the percentage difference between the estimated value of the quantity of interest to the asymptotic value. A refinement ratio (r) =2 and safety factor (SF) =1.25 was used as three mesh grades have been considered for the study. Supplementary Material Table 2 shown below summarises the calculations for Grid Convergence Index for Case 1 and detailed calculations can be found in attached .xls file ‘Calculations_Grid_Convergence_Index’. GCI_12_ is the GCI value calculated between the fine mesh and medium mesh and GCI_23_ is the GCI value calculated between medium and coarse mesh. It can be clearly seen that both GCI_12_ and GCI_23_ are less than 5% error and is considered acceptable for wall shear stress. Also, it was shown that the relation eq 1[1] was satisfied and verified that the study was conducted with mesh grades sufficiently fine for asymptotic mesh convergence.

$\mathrm{GCI}_{23}$ ${=r^{p}\mathrm{GCI}}_{12}$ (1)

where, p is the order of convergence and r is the refinement ratio

As a result, a medium mesh was considered sufficiently fine enough considering tradeoff between computational cost and accuracy of the solution obtained. The computational domain of the final mesh generated for Case 1 is shown in Supplementary Material Figure 3.

***Supplementary Material Table 1:*** Details of the mesh generated for Case 1

| **Mesh** | **Cell size (m)** | **Total number of cells (10^6^)** | **Wall shear stress (Pa)** |
| --- | --- | --- | --- |
| Coarse (Mesh 3) | 0.001 | 0.01008 | 0.09841 |
| Medium (Mesh 2) | 0.0005 | 0.04896 | 0.09369 |
| Fine (Mesh 1) | 0.00025 | 0.27648 | 0.09271 |
| Richardson Extrapolation |  |  | 0.09245 |


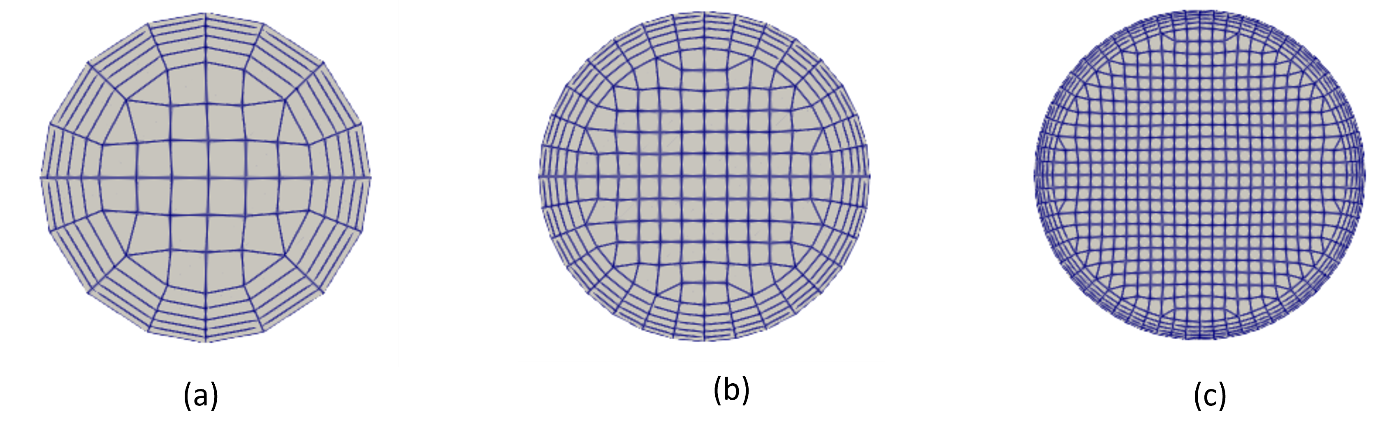


**Supplementary Material Figure 1:** Mesh generated for Case 1; (a) Coarse Mesh (Mesh 3); (b) Medium mesh (Mesh 2); Fine mesh (Mesh 3)


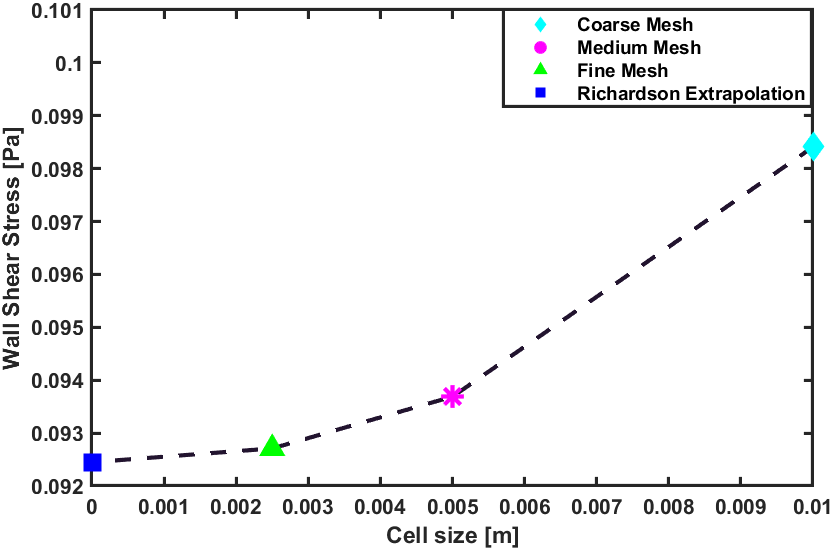


**Supplementary Material Figure 2:** Wall shear stress values for each mesh grade used for the mesh convergence study for Case 1

***Supplementary Material* *Table 2:*** Calculation of Grid Convergence Index (GCI ) for Case 1

| **Order of Convergence (p)** | **GCI_12_ (%)** | **GCI_23_ (%)** | **Safety Factor (SF)** | **Refinement ratio (r)** |
| --- | --- | --- | --- | --- |
| 2.26793 | 0.34622 | 1.65011 | 1.25 | 2 |


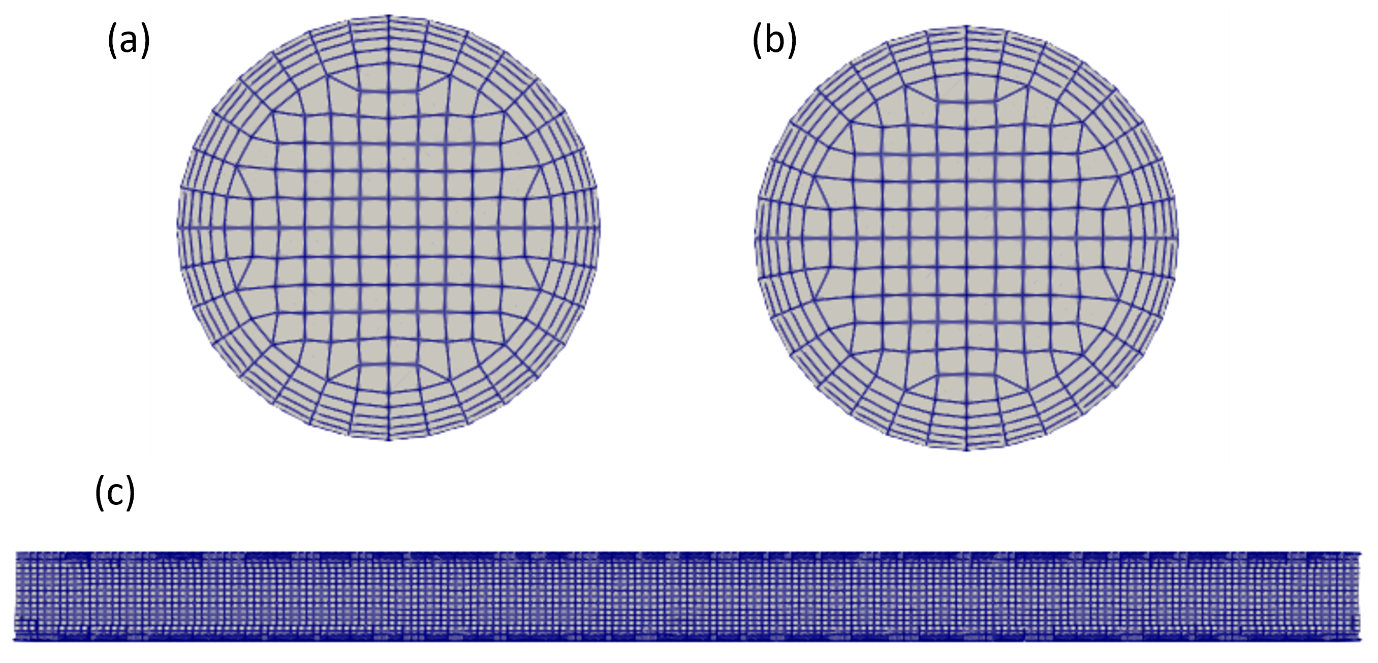


**Supplementary Material Figure 3:**Final mesh generated for the computational domain for Case 1; (a) Inlet; (b) Outlet; (c) Wall

**Case 2:** Outlet of the peristaltic pump to the pseudovessel outlet

The details of the mesh generated for Case 2 has been shown in Supplementary Material Table 3 and Supplementary Material Figure 4. The mesh was further refined at the interface of silicone tubing-connector (converging section) and connector-pseudovessel (sudden expansion). The plot shown in Supplementary Material Figure 5 demonstrates that as the mesh is refined further, the wall shear stress values approaches asymptotic convergence with Richardson extrapolation value obtained at zero cell size. A refinement ratio (r) =2 and safety factor (SF) =1.25 was used as three mesh grades have been considered for the study. Supplementary Material Table 4 shown below summarises the calculations for Grid Convergence Index for Case 2 and detailed calculations can be found in attached .xls file ‘Calculations_Grid_Convergence_Index’. GCI_12_ is the GCI value calculated between the fine mesh and medium mesh and GCI_23_ is the GCI value calculated between medium and coarse mesh. It can be clearly seen that both GCI_12_ and GCI_23_ are less than 5% error and is considered acceptable for wall shear stress. Similar to Case 1, it was shown that eq 1 was satisfied and verified that the study was conducted with mesh grades sufficiently fine for asymptotic mesh convergence. As a result, a medium mesh was considered sufficiently fine enough for the CFD model for Case 2 considering the trade-off between accuracy of the solution and computational time. The computational domain of the final mesh generated for Case 2 is shown in Supplementary Material Figure 6.

***Supplementary Material Table 3:*** Details of the mesh generated for Case 2

| Mesh | Cell Size (m) | Total Number of Cells (10^6^) | Wall shear stress (Pa) |
| --- | --- | --- | --- |
| Coarse (Mesh 3) | 0.001 | 0.032 | 0.09488 |
| Medium (Mesh 2) | 0.0005 | 0.201 | 0.08716 |
| Fine (Mesh 1) | 0.00025 | 1.225 | 0.08601 |
| Richardson Extrapolation |  |  | 0.08580 |


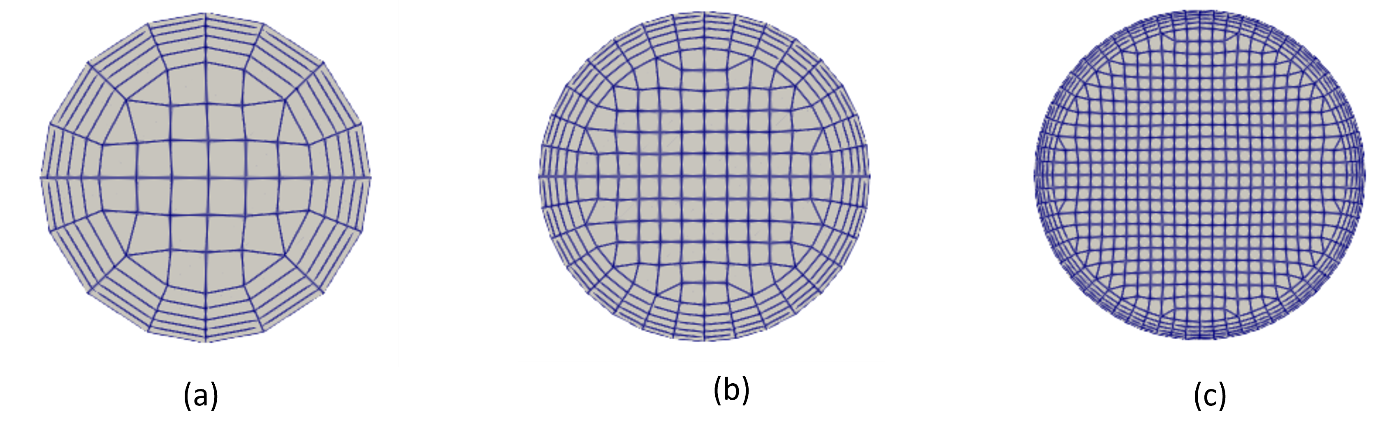


**Supplementary Material Figure 4:** Mesh generated for Case 2; (a) Coarse Mesh (Mesh 3); (b) Medium mesh (Mesh 2); Fine mesh (Mesh 3)


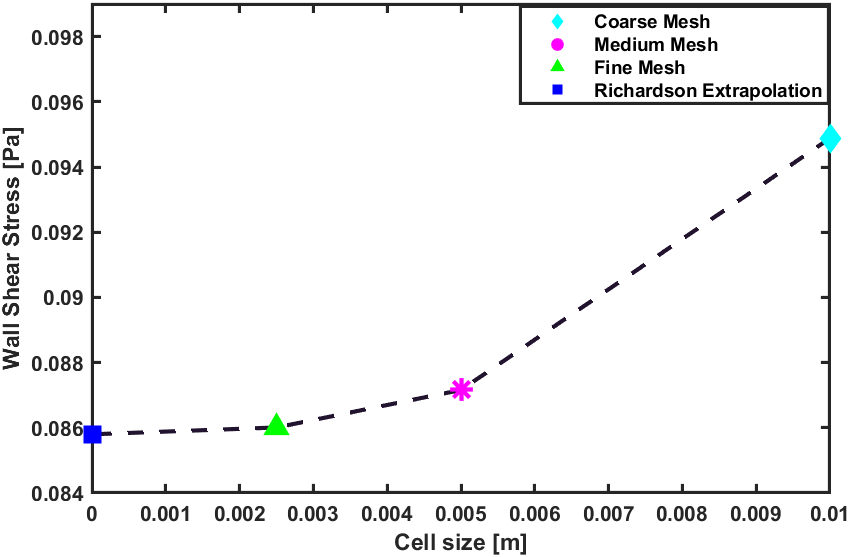


**Supplementary Material Figure 5:** Wall shear stress values for each mesh grade used for the mesh convergence study for Case 2

***Supplementary Material Table 4:*** Calculation of Grid Convergence Index (GCI ) for Case 2

| **Order of Convergence (p)** | **GCI_12_ (%)** | **GCI_23_ (%)** | **Safety Factor (SF)** | **Refinement ratio (r)** |
| --- | --- | --- | --- | --- |
| 2.74696 | 0.29254 | 1.93794 | 1.25 | 2 |


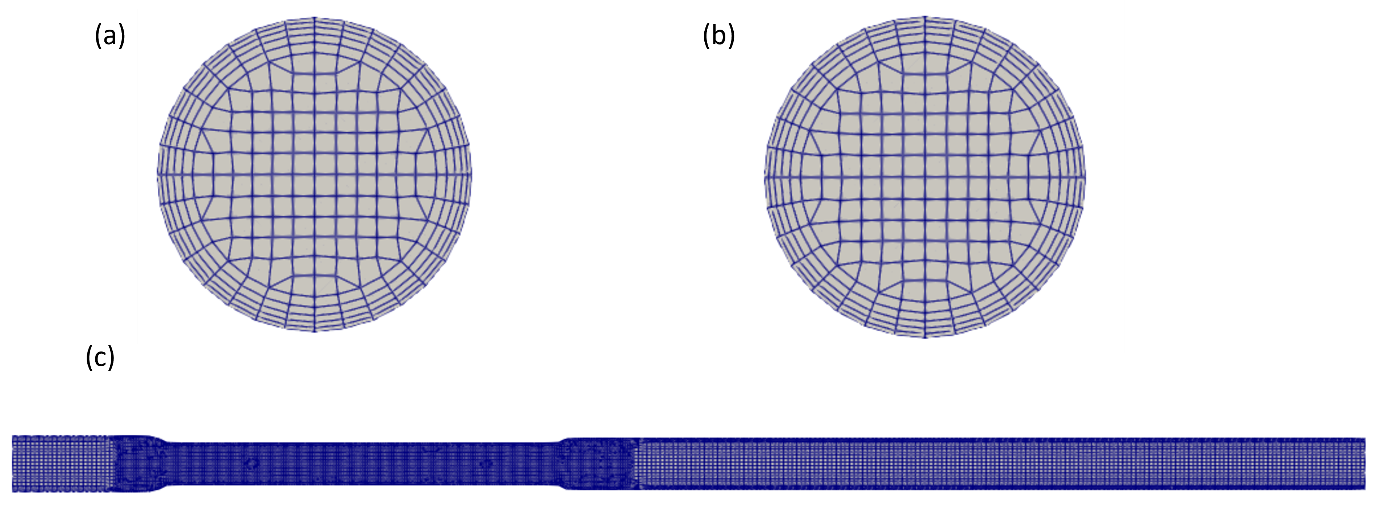


**Supplementary Material Figure 6:** Final mesh (medium mesh) generated for the computational domain for Case 2; (a) Inlet; (b) Outlet; (c) Wall

# EC Cell Orientation plots for Day 1 and Day 4 time point


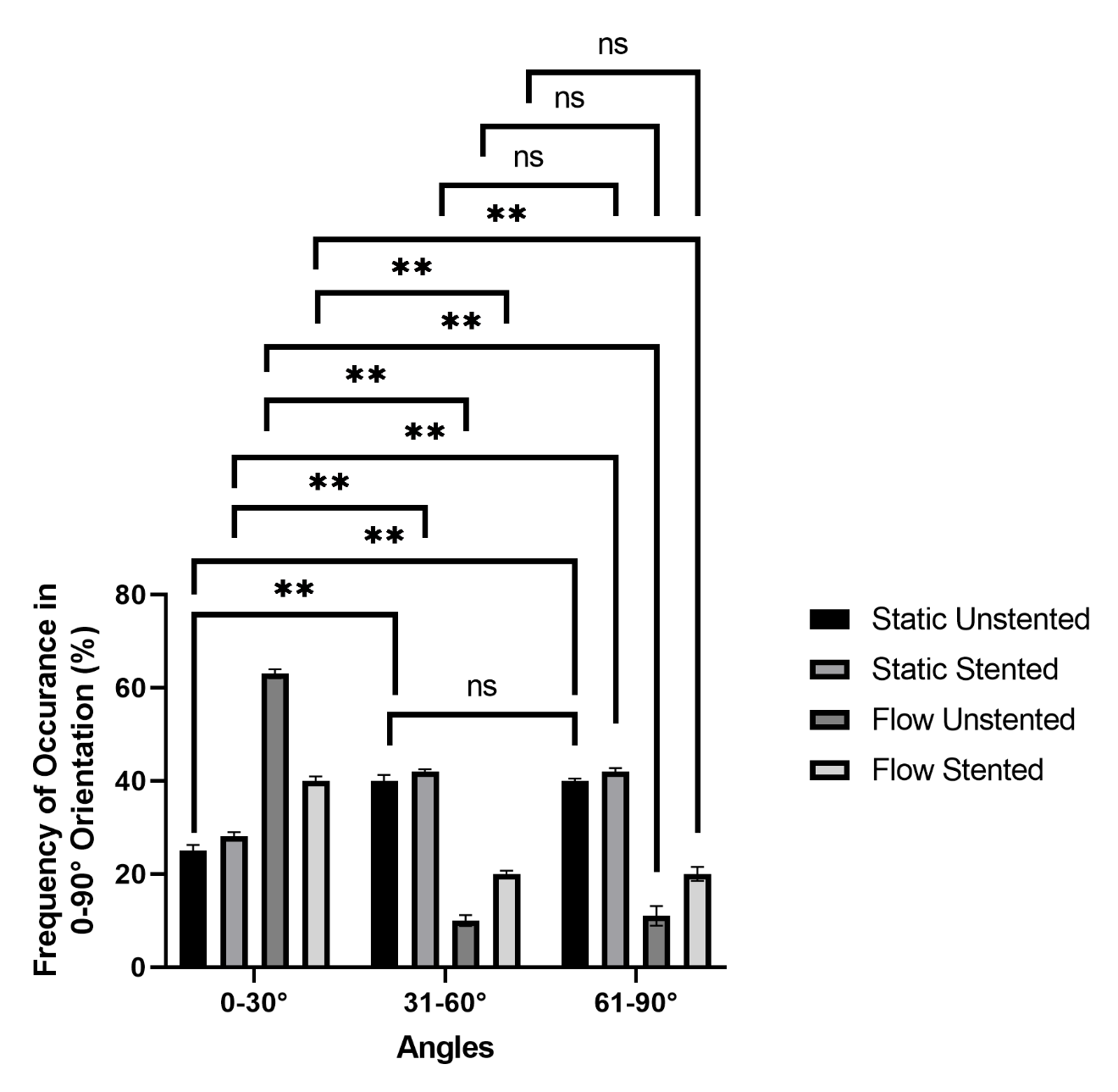
**Supplementary Figure 7:** EC cell orientation and frequency of occurrence at day 1 time point. Error bar represents mean ± S.D, * represents p < 0.05, ** represents p < 0.01 and ns being non-significant.


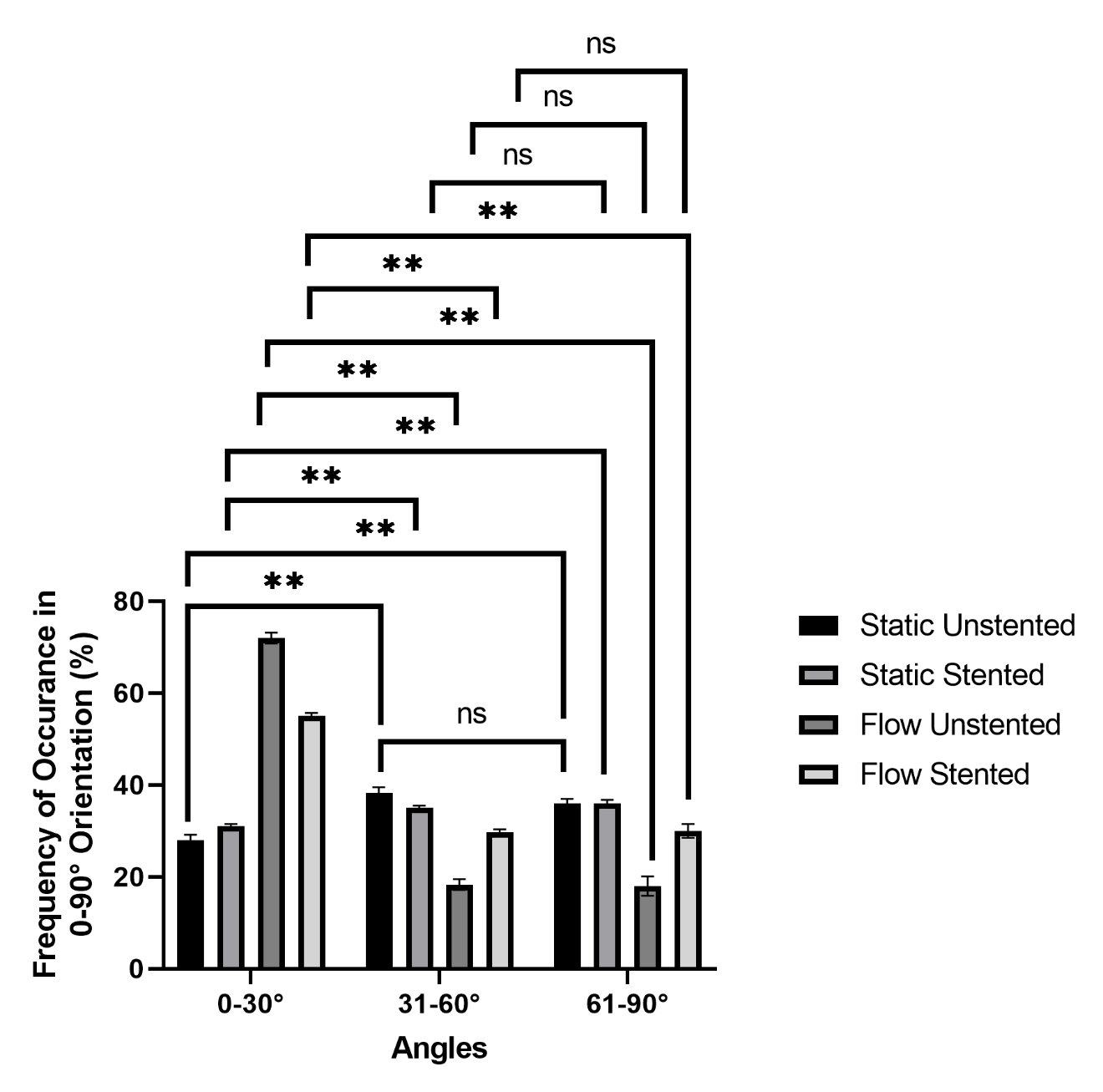


**Supplementary Figure 8:** EC cell orientation and frequency of occurrence at day 4 time point. Error bar represents mean ± S.D, * represents p < 0.05, ** represents p < 0.01 and ns being non-significant.
